# CSF contamination contributes to apparent microstructural alterations in mild cognitive impairment

**DOI:** 10.1016/j.neuroimage.2014.01.031

**Published:** 2014-05-15

**Authors:** Rok Berlot, Claudia Metzler-Baddeley, Derek K. Jones, Michael J. O'Sullivan

**Affiliations:** a Department of Clinical Neuroscience, Institute of Psychiatry, King's College London, 16 De Crespigny Park, London SE5 8AF, UK; b Department of Neurology, University Medical Centre Ljubljana, Zaloska 2, 1000 Ljubljana, Slovenia; c Cardiff University Brain Research Imaging Centre (CUBRIC), School of Psychology, Park Place, Cardiff CF10 3AT, UK; d The Neuroscience and Mental Health Research Institute, Cardiff University, Park Place, Cardiff CF10 3AT, UK

**Keywords:** Diffusion MRI, Partial volume effects, Atrophy, Mild cognitive impairment, Tractography, Tract-based spatial statistics

## Abstract

Diffusion MRI is used widely to probe microstructural alterations in neurological and psychiatric disease. However, ageing and neurodegeneration are also associated with atrophy, which leads to artefacts through partial volume effects due to cerebrospinal-fluid contamination (CSFC). The aim of this study was to explore the influence of CSFC on apparent microstructural changes in mild cognitive impairment (MCI) at several spatial levels: individually reconstructed tracts; at the level of a whole white matter skeleton (tract-based spatial statistics); and histograms derived from all white matter. 25 individuals with MCI and 20 matched controls underwent diffusion MRI. We corrected for CSFC using a post-acquisition voxel-by-voxel approach of free-water elimination. Tracts varied in their susceptibility to CSFC. The apparent pattern of tract involvement in disease shifted when correction was applied. Both spurious group differences, driven by CSFC, and masking of true differences were observed. Tract-based spatial statistics were found to be robust across much of the skeleton but with some localised CSFC effects. Diffusivity measures were affected disproportionately in MCI, and group differences in fornix microstructure were exaggerated. Group differences in white matter histogram measures were also partly driven by CSFC. For diffusivity measures, up to two thirds of observed group differences were due to CSFC. Our results demonstrate that CSFC has an impact on quantitative differences between MCI and controls. Furthermore, it affects the apparent spatial pattern of white matter involvement. Free-water elimination provides a step towards disentangling intrinsic and volumetric alterations in individuals prone to atrophy.

## Highlights

• We examine the effect of CSFC on perceived microstructural alterations in MCI.
• We correct for CSFC with free-water elimination and assess at three spatial levels.
• The pattern of individual tract involvement is shifted due to CSFC.
• TBSS is robust to CSFC in much of the skeleton.
• Group differences in white matter diffusion histograms are partly driven by CSFC.

## Graphical abstract

**Figure:**
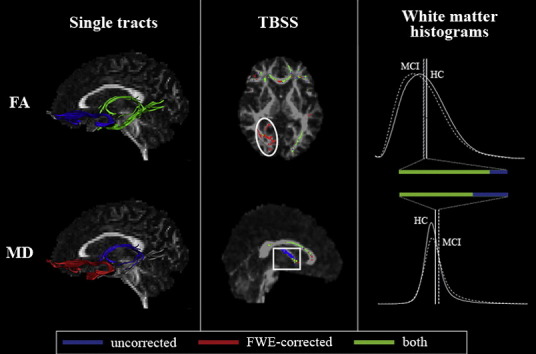

## Introduction

Diffusion MRI has provided evidence of white matter microstructural alterations in ageing and neurodegeneration, as well as developmental and psychiatric conditions in which the underlying pathology remains elusive. Most often, these alterations are interpreted in terms of *intrinsic* tissue microstructure. However, these measures are only structure-specific insofar as a voxel does not contain heterogeneous fibre populations or tissues. Partial volume averaging of MRI signal between the cerebrospinal fluid (CSF) and the underlying brain parenchyma is a particular problem due to vastly different diffusion properties of water molecules in these compartments. Inclusion of CSF in a voxel results in an increase of isotropic diffusivity, generally leading to an overestimation of mean (MD), axial (AD) and radial diffusivity (RD) and decreased values of fractional anisotropy (FA) (Madden et al., 2012).

Partial volume effects (PVE) can confound the results of diffusion MRI studies because of tissue loss or atrophy. The degree of brain atrophy affects the amount of CSF partial voluming as decreasing volume of white matter structures has been shown to increase the relative contribution of PVE-contaminated voxels due to an increase in surface area to volume ratio (Vos et al., 2011). This is most marked in areas where the white matter abuts immediately to CSF filled spaces. PVE are a particularly relevant source of bias in ageing and degenerative states, which display alterations in size and shape of white matter tracts due to atrophy in addition to changes in their intrinsic diffusion properties. In Alzheimer's disease, for example, both diffusion MRI changes in brain parenchyma and macroscopic atrophy have been demonstrated consistently in a number of studies. Even in the recognised prodromal phase, mild cognitive impairment, subtle volumetric alterations are established (Han et al., 2012). Therefore, it seems likely that some degree of volumetric change frequently coexists with microstructural change (Sullivan and Pfefferbaum, 2011).

A number of strategies have been used to attempt to correct for the effects of atrophy-related PVE and cerebrospinal-fluid contamination (CSFC). The simplest is to use whole brain volumetric measures as a covariate (e.g. Rashid et al., 2004; Takao et al., 2011). However, when dealing with measurements from single tracts of interest, a whole brain volumetric measure fails to capture the local CSF environment and performs poorly for some tracts (Metzler-Baddeley et al., 2012a). Other studies have attempted to tackle local variations in PVE on a voxel-by-voxel basis by using voxel tissue densities as a covariate (Canu et al., 2010). However, the amount of CSFC does not reflect only the intravoxel volumetric ratio of CSF and brain tissue. The contribution of CSF is accentuated due to its higher proton density, T_2_ relaxation time and diffusion coefficient (Papadakis et al., 2002). Therefore, the measured diffusivity is not simply a weighted mean of the tissue and CSF diffusion coefficients, which makes the general linear model-based approach of accounting for CSFC suboptimal.

The Free Water Elimination method (Pasternak et al., 2009) addresses the problem by modelling the effect of CSFC on intravoxel diffusion data directly. This is done by adopting a two compartment model and fitting two tensors to diffusion data, one anisotropic and one isotropic with diffusion characteristics of free water (Pierpaoli and Jones, 2004). By adding the constraint of neighbouring voxels having smooth variation of diffusion tensor, Pasternak et al. (2009) have developed this approach into a post-acquisition method of free-water elimination (FWE), which can be employed in data acquired by typical single b-value acquisition protocols. A by-product of FWE is that it provides a voxelwise map of *tissue volume fraction*, which might provide complementary information on tract structure attributable to atrophy at a microstructural scale (Metzler-Baddeley et al., 2012b).

In a previous study we tested the FWE approach in ageing using the fornix as an exemplar (Metzler-Baddeley et al., 2012a). The fornix was chosen because it is both a critical tract in terms of memory and also uniquely susceptible to CSFC due to its intraventricular location. FWE distinguished true microstructural change from CSFC effects on fornix diffusion measurements, in a way that was consistent with mathematical simulations as well as with recent high spatial resolution DTI measurements in the fornix (Sakaie et al., 2013). The purpose of the current study was to explore the effect of CSFC on apparent microstructural alterations in disease by investigating Mild Cognitive Impairment (MCI), the prodromal form of Alzheimer's disease. The MCI population was investigated as we were interested in the effect even subtle atrophy due to neurodegeneration might have on CSF-based PVE. The effect of CSFC was studied at three spatial levels, often used in clinical studies: analysis of individual tracts; analysis at the level of a white matter skeleton (tract-based spatial statistics); and whole white matter histograms.

## Methods

### Participants

25 patients with MCI were recruited from the Cardiff Memory Clinic. All patients underwent standardised assessment including clinical history, ascertainment of vascular risk factors, neurological examination, basic haematology and biochemistry investigations, neuroimaging with CT or MRI and cognitive screening with the Addenbrooke's Cognitive Examination (Mioshi et al., 2006). Diagnosis of MCI was based on established current criteria (Albert et al., 2011). Objective memory impairment was confirmed by a score of > 1.5 SDs below age-matched controls on either the Addenbrooke's verbal memory subscore or the visual memory test from the Repeatable Battery for the Assessment of Neurological Status. All patients had a Mini-Mental State Examination score of ≥ 24 (mean 26, SD 1.7) and a Clinical Dementia Rating of 0.5 (Morris, 1993). 46 healthy elderly individuals were recruited, as controls, from the local community by advertisements and via a Cardiff University Community Panel of healthy research volunteers.

Exclusion criteria for both groups were: previous moderate to severe head injury; prior or current alcohol and/or drug abuse (as defined by *DSM-IV-TR*); previous disabling or large-artery stroke or cerebral haemorrhage; known peripheral, cervical, or coronary artery disease; structural heart disease or heart failure; and contraindications to MRI. In addition, no patient with MCI met diagnostic criteria or had characteristic cognitive or behavioural features to suggest other degenerative disorders. An additional exclusion criterion for healthy participants was the presence of subjective memory symptoms either in the past or currently. This assessment was based on a questionnaire the participants filled in about their past medical history. All participants were also evaluated with a detailed neuropsychological battery, as detailed in Metzler-Baddeley et al. (2011). Healthy participants were free of evidence of impairment on this battery.

From the sample of healthy older volunteers, 20 healthy control participants were selected to match the MCI group: participants older than 65 years (all MCI participants were older than 65) and with a verbal IQ not exceeding two SDs above the average patient IQ in the National Adult Reading Test-Revised (NART-R) (Nelson and Willison, 1991) were included. The groups were matched for age (MCI 76.8 ± 7.3 years, controls 74.0 ± 6.5 years, *p* = .19), education (MCI 14 ± 4 years, controls 15 ± 3 years, *p* = .08) and estimated premorbid IQ (MCI 115 ± 11, controls 120 ± 9, *p* = .08). 11 of the MCI group and 10 of the control group were female. Further clinical and neuropsychological details can be found in Metzler-Baddeley et al. (2012b).

Ethical approval for the study was provided by the South East Wales Research Ethics Commitee (panel C). All participants provided informed, written consent.

### Image acquisition

Diffusion-weighted MRI data were acquired using a 3T GE HDx MRI system (General Electric) with a twice-refocused spin-echo echo planar imaging sequence, providing whole oblique axial (parallel to the commissural plane) brain coverage. Data acquisition was peripherally gated to the cardiac cycle. Data were acquired for 60 slices of 2.4 mm thickness, with a field of view of 23 cm and an acquisition matrix of 96 × 96. Echo delay time was 87 ms and parallel imaging (array spatial sensitivity encoding (ASSET) factor = 2) was used. The b-value was 1200 s/mm^2^. In each imaging session, data were acquired with diffusion encoded along 30 isotropically distributed directions and 3 non-diffusion-weighted scans, according to an optimised gradient vector scheme. Acquisition time was approximately 13 min.

T1-weighted structural MRI data were acquired using a 3D fast spoiled gradient recalled (FSPGR) echo sequence, acquired with a matrix of 256 × 256 × 176 and field of view of 256 × 256 × 176 mm, resulting in isotropic (1 mm) resolution. The timing parameters were TR/TE/TI = 7.9/3.0/450 ms, and the flip angle was 20°.

### Image processing

The acquired diffusion-weighted images were corrected for distortions, introduced by diffusion-weighting gradients, and for motion artefacts. This was achieved using a global affine registration of each image volume to the first non-diffusion weighted volume, using normalised mutual information as the cost-function, followed by appropriate re-orientation of the encoding vectors (Leemans and Jones, 2009) and modulation of the signal intensity by the Jacobian determinant of the transformation (Jones and Cercignani, 2010).

A single-tensor model was fitted to the data in each voxel (Basser et al., 1994) and uncorrected values of fractional anisotropy (FA^u^), mean diffusivity (MD^u^), axial diffusivity (AD^u^) and radial diffusivity (RD^u^) were computed. Additionally, a two compartment model using the FWE approach was fitted to the data and corrected values FA^c^, MD^c^, AD^c^ and RD^c^ were computed (Pasternak et al., 2009).

### Tractography and single tracts

Tractography was performed using ExploreDTI (www.exploreDTI.com) (Leemans et al., 2009). Whole-brain tractography was performed using every voxel as a seed point. To assess measures of the fornix, deterministic tracking based on constrained spherical harmonic deconvolution (CSD) (Jeurissen et al., 2011; Tournier et al., 2004) was used instead of the tensor model as a more appropriate technique because of the proximity of other white matter tracts to the fornix. A deterministic tracking algorithm estimated the principal diffusion orientation at each seed point and propagated in 0.5 mm steps along this direction. The fibre orientations were then estimated at the new location and tracking moved a further 0.5 mm along the direction that subtended the smallest angle to the current trajectory. A pathway was traced through the data until the fibre orientation density function peak fell below 0.1 or the direction of the pathway changed through an angle greater than 60°.

Three-dimensional reconstructions of the fornix, uncinate fasciculus (UNC) and parahippocampal cingulum (PHC) were then extracted by applying multiple waypoint region-of-interest (ROI) masks and Boolean logical operators, as detailed in Supplementary material and in previous publications (Metzler-Baddeley et al., 2011, 2012b). All ROIs were manually drawn for each individual dataset in native space on colour-coded fibre orientation maps by a single blinded operator (CMB), using landmark techniques that have previously been shown to be highly reproducible. The metrics of FA^u^, FA^c^, MD^u^ and MD^c^ were sampled at each 0.5 mm step along the pathways, providing tract-specific means.

### Tract-based spatial statistics

Voxelwise statistical analysis of diffusion data was carried out using TBSS (Tract-Based Spatial Statistics) (Smith et al., 2006), part of FSL (FMRIB Software Library, http://www.fmrib.ox.ac.uk/fsl/, Version 5.0) (Smith et al., 2004). A representative FA map was selected automatically from the pooled dataset and its uncorrected and FWE-corrected versions were used as study-specific templates for the uncorrected and corrected dataset, respectively. A mean FA skeleton was determined after aligning the FA maps first to the appropriate target image and then into MNI152 standard space. Each subject's aligned FA data were then projected to the skeleton. The TBSS script involves masking the mean FA image by the non-zero FA voxels common to all participants in order to compare only the skeleton voxels that are present in all subjects. Fitting the two-compartment model resulted in all voxels outside of the brain having very low but non-zero estimates of diffusion indices. To remove these voxels with spurious near zero values in corrected maps, each participant's FWE-corrected FA map was masked by the corresponding FA^u^ map. Voxel projections defined in this way were applied to project both uncorrected and FWE-corrected MD, AD and RD values to the white matter skeleton. Additionally, maps of tissue volume fraction (*f*) were used to generate voxelwise group comparisons.

To separate the influence of CSF elimination from any possible registration changes induced by the FWE approach, analysis was repeated using identical spatial transformations for both datasets.

Locations of regions displaying microstructural alterations were determined by manual comparison with the John Hopkins University ICBM-DTI-81 White Matter Labels atlas (Mori et al., 2005), provided within FSL.

### White matter diffusion histograms

Acquired FWE-corrected and uncorrected FA maps were coregistered with each subject's T1-weighted image with an affine transformation in FLIRT (FMRIB's Linear Image Registration Tool) (Jenkinson et al., 2002). The same transformations were applied to each subject's FWE-corrected and uncorrected MD, AD and RD maps. Registrations were visually checked according to callosal landmarks to ensure good alignment (landmark discrepancy of less than 3 mm).

Non-brain tissue was removed from T1-weighted images using BET2 (Brain Extraction Tool) (Smith, 2002). Images were then segmented into grey matter, white matter and CSF using the binary segmentation option in FAST (FMRIB's Automated Segmentation Tool) (Zhang et al., 2001). The white matter masks were created and applied to the T1-aligned FA^u^, MD^u^, AD^u^, RD^u^, FA^c^, MD^c^, AD^c^ and RD^c^ maps.

Histograms were calculated for both uncorrected and FWE-corrected FA, MD, AD and RD maps of white matter (FA: range 0.000–1.000, bin width 0.005; MD, AD and RD: range 0.10–3.00, bin width 0.01 · 10^− 3^mm^2^ s^− 1^). To account for individual differences in brain size, bins were normalised by dividing the height of each bin count by the total number of voxels contributing to the histogram.

### Statistical analysis

Unpaired t-tests were used to compare individual tracts between control and MCI groups. Participants in whom we were unable to reconstruct one or more of the tracts reliably were excluded from the comparison for that tract (the left PHC and right PHC were each missing in two patients with MCI). Repeated unpaired t-tests were preferred as this method most closely recapitulates the sort of group analysis that would be performed in practice in clinical case–control studies. To provide a statistical comparison between uncorrected and FWE-corrected results and to account for the impact FWE might have on the variance of the data, an additional analysis with two-way mixed-design ANOVA was also performed (with participant group as independent measure and uncorrected/FWE-corrected tract parameter value as repeated measure).

Unpaired t-tests were also employed to compare voxelwise maps across MCI and controls and were performed separately for uncorrected and FWE-corrected diffusion maps. To determine how the contribution of PVE differed in the two groups, an unpaired *t*-test was also performed to compare difference images, obtained after subtracting the corrected skeletonised parameter maps from the uncorrected ones throughout parts of the mean FA skeleton that were common to both datasets. This method offers the nearest approximation to a repeated-measure ANOVA, which is not easily implemented in TBSS where statistical inference is based on permutation. For all analyses, five thousand permutations were performed using *randomise* software (Nichols and Holmes, 2002). Resulting statistical maps were thresholded for *p* < .05, correcting for multiple comparisons using threshold-free cluster enhancement (TFCE) (Smith and Nichols, 2009).

To analyse group differences in diffusion histograms, mean parameter value, histogram peak location (location of the modal value) and peak height frequency (proportion of voxels at modal value) were calculated from each histogram. Simple independent group t-tests were performed to assess for group differences in the uncorrected and corrected datasets. A two-way mixed-design ANOVA analysis was also performed with participant group as independent measure and uncorrected/FWE-corrected histogram parameter value as repeated measure.

## Results

### Tractography and single tracts

Group comparisons based on both corrected and uncorrected measurements are given in Table 1. Tracts for which correction led to a different outcome are highlighted. Examples were seen of both spurious significant differences driven by CSFC (fornix MD and left uncinate FA) and the masking of a true difference by CSFC (left and right uncinate MD).

Fornix FA demonstrated a significant group difference in both the corrected and uncorrected analyses. The comparison shows that the relative difference between groups was greater in FWE-corrected data: correcting for CSFC seemed to unmask 25% of additional intrinsic microstructural alteration. In both analyses, the left and right PHC showed *increased* values of FA in MCI. This difference was exaggerated in the uncorrected analysis due to CSFC (15% of the relative group difference for the left and 21% for the right PHC).

Repeated-measures ANOVA analysis revealed a significant main effect of FWE for all assessed metrics (see Inline Supplementary Table S1). There was a significant interaction of FWE procedure and participant group membership for fornix FA and MD.

Repeated-measures ANOVA analysis revealed a significant main effect of FWE for all assessed metrics (see Inline Supplementary Table S1). There was a significant interaction of FWE procedure and participant group membership for fornix FA and MD.

Inline Supplementary Table S1

**Table S1:** Comparison of individual tracts between MCI and controls, before and after correction with Free Water Elimination, with a two-way mixed-design ANOVA. Tract-specific average values (SD) of fractional anisotropy (FA, no unit) and mean diffusivity (MD, 10^− 3^ mm^2 ^s^− 1^) are provided. Abbreviations: UNC, uncinate fasciculus; PHC, parahippocampal cingulum; MCI, mild cognitive impairment; HC, healthy control; FWE, Free Water Elimination; *F* is the F-test statistic and *p* the associated probability.

			Uncorrected	FWE-corrected	Within-subjects contrast (FWE)		Within-subjects contrast (FWExGroup)		Between-subjects effect (Group)	
			Mean (SD)	Mean (SD)	*F*	*p*	*F*	*p*	*F*	*p*
Fornix	FA	MCI	0.201 (0.027)	0.237 (0.045)						
		HC	0.224 (0.023)	0.272 (0.037)	183.788	< .001***	4.243	.046*	8.546	.006**
	MD	MCI	1.876 (0.238)	1.020 (0.125)						
		HC	1.660 (0.205)	1.065 (0.069)	315.976	< .001***	10.249	.003**	6.816	.012*
UNC left	FA	MCI	0.401 (0.025)	0.429 (0.053)						
		HC	0.377 (0.030)	0.418 (0.029)	38.262	< .001***	1.371	.248	3.239	.079
	MD	MCI	0.880 (0.048)	0.802 (0.040)						
		HC	0.861 (0.043)	0.781 (0.027)	415.116	< .001***	.069	.793	2.983	.091
UNC right	FA	MCI	0.395 (0.027)	0.427 (0.056)						
		HC	0.380 (0.023)	0.420 (0.025)	35.287	< .001***	.441	.510	1.319	.257
	MD	MCI	0.892 (0.036)	0.816 (0.047)						
		HC	0.872 (0.035)	0.790 (0.023)	220.592	< .001***	.450	.506	5.580	.023*
PHC left	FA	MCI	0.342 (0.033)	0.386 (0.032)						
		HC	0.311 (0.023)	0.356 (0.023)	1331.118	< .001***	.029	.865	12.050	.001**
	MD	MCI	0.845 (0.054)	0.786 (0.040)						
		HC	0.846 (0.042)	0.785 (0.028)	453.435	< .001***	.029	.866	.001	.970
PHC right	FA	MCI	0.364 (0.037)	0.409 (0.033)						
		HC	0.331 (0.026)	0.379 (0.026)	611.395	< .001***	.380	.541	11.522	.002**
	MD	MCI	0.827 (0.067)	0.767 (0.042)						
		HC	0.829 (0.048)	0.772 (0.031)	202.064	< .001***	.095	.759	.054	.818

Significance: *p < .05, **p < .01, ***p < .001.

Inline Supplementary Table S1

Inline Supplementary Table S1 can be found online at http://dx.doi.org/10.1016/j.neuroimage.2014.01.031.

### Tract-based spatial statistics

**Fig. 1:**
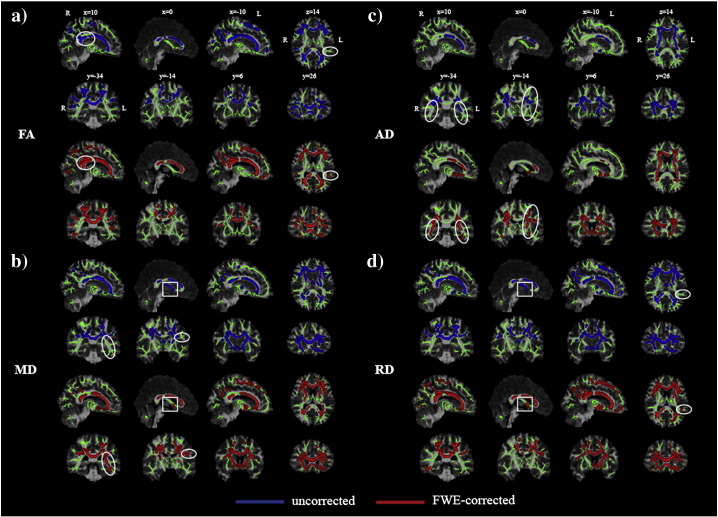
Group differences based on tract-based spatial statistics, before (dark grey; blue in online version) and after (white; red in online version) correction of images by Free Water Elimination. a) Results for reduced fractional anisotropy (FA); b) increased mean diffusivity (MD); c) increased axial diffusivity (AD); d) increased radial diffusivity (RD). Statistical maps are corrected for family-wise error and thresholded for *p* < .05. They are overlaid on the registration target brain, transformed into MNI152 space. Box: fornix microstructural differences due to partial volume effect. Circle: group differences observed only in the corrected analysis.

**Fig. 2:**
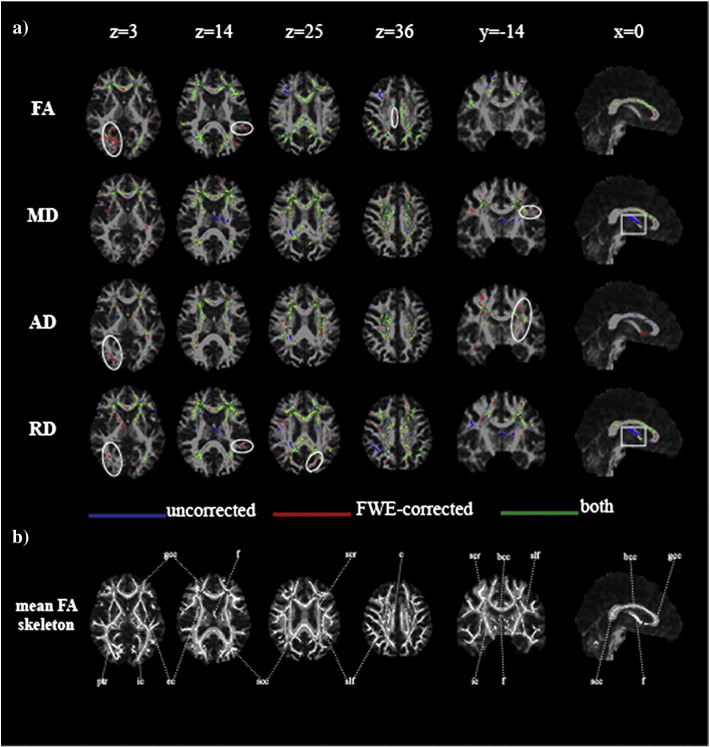
Group differences before and after Free Water Elimination superimposed on a common target image. a) Mean FA skeleton voxels showing significantly lower value of fractional anisotropy (FA) and higher values of mean (MD), axial (AD) and radial diffusivity (RD) in patients with mild cognitive impairment only in uncorrected dataset (dark grey; blue in online version), only after applying the free-water elimination (FWE) correction for CSF contamination (white; red in online version) or in both datasets (light grey; green in online version). Displayed results are corrected for family-wise error and thresholded for *p* < .05. Box: fornix microstructural differences due to partial volume effect. Circle: group differences observed only in the corrected analysis. b) Mean FA skeleton with labels according to JHU ICBM-DTI-81 White-Matter Labels atlas (bcc—body of corpus callosum, c—cingulum, ec—external capsule, f—fornix, gcc—genu of corpus callosum, ic—internal capsule, ptr—posterior thalamic radiation, scc—splenium of corpus callosum, scr—superior corona radiata, slf—superior longitudinal fasciculus). Statistical maps are overlaid on the registration target brain transformed into MNI152 space. Images are displayed in radiological view.

Fig. 1 shows maps of significant group differences for analyses based on both uncorrected and corrected data. Widespread areas of decreased FA and increased MD, AD and RD were found in both uncorrected and corrected TBSS analyses. No areas of increased FA or reduced diffusivity in MCI reached significance in either analysis. To check that observed differences were not due to minor inconsistencies in registration of corrected or uncorrected images, the analyses were repeated using identical spatial transformations for both datasets (Fig. 2). The effects of CSFC described below and emphasized in the figures were consistent across both analyses.

Although the results of the uncorrected and FWE-corrected analyses are broadly similar, closer inspection reveals localised differences, seen most clearly when the results are superimposed (Fig. 2). CSFC contributed to observed differences in fornix microstructure in uncorrected analyses (box, Figs. 1 and 2). This effect was most notable for MD and RD. In contrast, differences in the posterior thalamic radiation, cingulum and peripheral parietal and occipital white matter were demonstrated more clearly in the corrected analysis (circle, Figs. 1 and 2).

**Fig. 3:**
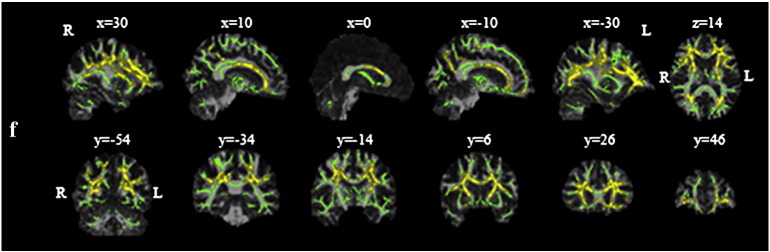
Group differences in tissue volume fraction (*f*). Regions of significant reduction of *f* in MCI are shown in white (yellow in online version). The background skeleton is shown in dark grey (green in online version). Displayed results are corrected for family-wise error and thresholded for *p* < .05. Statistical maps are overlaid on the registration target brain transformed into MNI152 space.

Maps of *f* – the tissue volume fraction, which is essentially a measure of microscopic atrophy – were also generated from the FWE approach. A TBSS analysis using *f* demonstrated significant differences between groups (Fig. 3). Interestingly, the spatial distribution of alterations in *f* was distinct from that found for other measures and also did not coincide with areas exhibiting inconsistencies between uncorrected and FWE-corrected analyses.

**Fig. 4:**
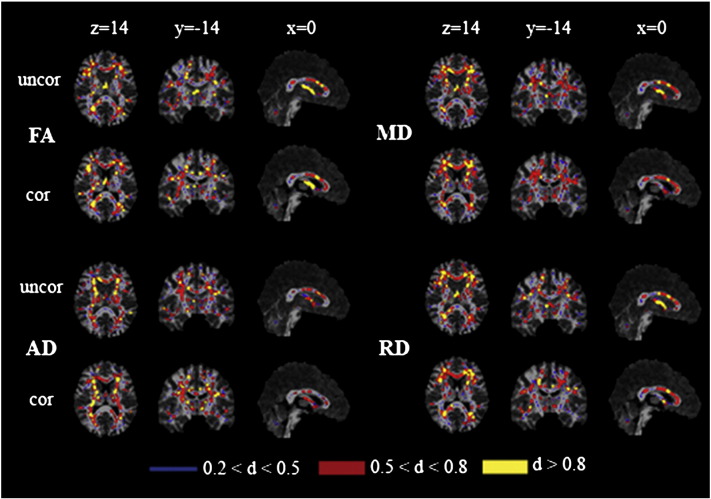
Effect sizes before and after Free Water Elimination. Maps of Cohen's *d* for differences in the values of fractional anisotropy (FA), mean (MD), axial (AD) and radial diffusivity (RD) in patients with mild cognitive impairment compared to controls throughout the mean FA skeleton for the uncorrected dataset (uncor) and after correcting for partial volume effect (cor). Effect sizes are displayed according to their values—small (thin white line, blue in online version), medium (thick white line, red in online version) and large (thick dark grey line, yellow in online version). Statistical maps are overlaid on the registration target brain transformed into MNI152 space. Images are displayed in radiological view.

The spatial pattern of effect of CSFC on measures of microstructure was also compared across groups (Suppl. Fig. 2). In MCI, a greater CSFC was found for diffusivity measurements in the genu of the corpus callosum, adjacent frontal lobe white matter, superior corona radiata and regions of parietal white matter. No areas exhibited greater CSFC in the control group. The effect of CSFC on FA measures did not differ between groups. This differential effect was also apparent in maps of effect size based on Cohen's *d* (Fig. 4). Effect size showed a marked reduction in the area of the fornix after applying the FWE approach for MD, RD and, to a lesser extent, AD. There is a shift towards larger effect sizes after correcting for CSFC for FA.

The spatial pattern of effect of CSFC on measures of microstructure was also compared across groups (Suppl. Fig. 2). In MCI, a greater CSFC was found for diffusivity measurements in the genu of the corpus callosum, adjacent frontal lobe white matter, superior corona radiata and regions of parietal white matter. No areas exhibited greater CSFC in the control group. The effect of CSFC on FA measures did not differ between groups. This differential effect was also apparent in maps of effect size based on Cohen's *d* (Fig. 4). Effect size showed a marked reduction in the area of the fornix after applying the FWE approach for MD, RD and, to a lesser extent, AD. There is a shift towards larger effect sizes after correcting for CSFC for FA.

### Whole white matter histograms

As expected, FA histograms were shifted to the left in patients with MCI and diffusivity histograms shifted to the right compared to healthy older volunteers. A similar qualitative pattern was retained after correcting for CSFC. Although both approaches yielded significant group differences (Table 2), comparison of metrics derived from the histograms suggested that a proportion of observed relative differences between groups was due to PVE. This proportion was greater for MD than for FA (33% versus 16% of the difference in mean values, respectively), and largest for AD (67%).

There was a significant main effect of FWE for all histogram parameters (see Inline Supplementary Table S2). For mean histogram values of MD, AD and RD and for MD histogram peak height, there was a significant interaction between FWE correction and participant group membership.

There was a significant main effect of FWE for all histogram parameters (see Inline Supplementary Table S2). For mean histogram values of MD, AD and RD and for MD histogram peak height, there was a significant interaction between FWE correction and participant group membership.

Inline Supplementary Table S2

**Table S2:** Summary statistics derived from histograms of MCI and control groups and results of a two-way mixed-design ANOVA. Mean and modal values of the histogram distribution for fractional anisotropy (FA, no unit), mean diffusivity (MD, 10^− 3^ mm^2 ^s^− 1^), axial diffusivity (AD, 10^− 3^ mm^2 ^s^− 1^) and radial diffusivity (RD, 10^− 3^ mm^2 ^s^− 1^) and corresponding histogram peak heights (proportion of voxels at modal value) for patients with mild cognitive impairment (MCI) and healthy elderly controls (HC) in the uncorrected dataset, and after correcting for partial volume effect using the free-water elimination (FWE) approach. *F* is the F-test statistic, *p* is the associated probability.

		Group	Uncorrected	FWE-corrected	Within-subjects contrast (FWE)		Within-subjects contrast (FWE × Group)		Between-subjects effect (Group)	
			Mean (SD)	Mean (SD)	*F*	*p*	*F*	*p*	*F*	*p*
FA	Mean	MCI	0.323 (0.029)	0.353 (0.028)	1270.361	< .001***	2.604	.114	8.861	.005**
		HC	0.345 (0.017)	0.373 (0.015)						
	Mode	MCI	0.262 (0.039)	0.284 (0.062)	38.348	< .001***	1.948	.170	12.089	.001**
		HC	0.300 (0.034)	0.336 (0.038)						
	Peak Height	MCI	0.0145 (0.0015)	0.0130 (0.0020)	182.138	< .001***	0.308	.582	1.347	.252
		HC	0.0139 (0.0009)	0.0127 (0.0008)						
MD	Mean	MCI	0.871 (0.066)	0.780 (0.039)	620.202	< .001***	5.536	.023*	5.471	.024*
		HC	0.833 (0.033)	0.757 (0.021)						
	Mode	MCI	0.803 (0.040)	0.753 (0.029)	624.846	< .001***	1.220	.275	3.823	.057
		HC	0.784 (0.030)	0.737 (0.020)						
	Peak Height	MCI	0.0340 (0.0070)	0.0529 (0.0107)	1662.902	< .001***	10.848	.002**	13.767	.001**
		HC	0.0411 (0.0053)	0.0634 (0.0079)						
AD	Mean	MCI	1.166 (0.055)	1.071 (0.029)	638.238	< .001***	4.909	.032*	2.016	.163
		HC	1.143 (0.031)	1.064 (0.018)						
	Mode	MCI	1.068 (0.042)	0.997 (0.031)	712.148	< .001***	.633	.431	1.569	.217
		HC	1.059 (0.029)	0.983 (0.019)						
	Peak Height	MCI	0.0196 (0.0044)	0.0249 (0.0022)	125.521	< .001***	2.769	.103	7.596	.009**
		HC	0.0223 (0.0014)	0.0262 (0.0013)						
RD	Mean	MCI	0.723 (0.073)	0.634 (0.046)	607.273	< .001***	5.377	.025*	6.927	.012**
		HC	0.677 (0.035)	0.604 (0.024)						
	Mode	MCI	0.661 (0.045)	0.618 (0.033)	397.796	< .001***	3.808	.058	3.899	.055
		HC	0.637 (0.033)	0.601 (0.025)						
	Peak Height	MCI	0.0285 (0.0048)	0.0370 (0.0048)	3523.479	< .001***	.149	.702	11.606	.001**
		HC	0.0326 (0.0027)	0.0410 (0.0028)						

Significance: *p < .05, **p < .01, ***p < .001.

Inline Supplementary Table S2

Inline Supplementary Table S2 can be found online at http://dx.doi.org/10.1016/j.neuroimage.2014.01.031.

## Discussion

Correction for CSF contamination with the free-water elimination approach had a significant influence on apparent group differences between MCI and controls. The most obvious effect was on the pattern of individual tract involvement but localised differences in tract-based spatial statistics and a quantitative influence on whole white matter histograms were also observed. At the level of individual tracts, both spurious group differences, driven by CSFC, and masking of true differences by CSFC were observed. Maps of significant differences from tract-based spatial statistics were robust to CSFC across much of the skeleton but illustrated local differences, most notably in the region of the fornix. The effect on histograms depended on the chosen measure and was largest for histograms based on diffusivity, for which up to two thirds of the quantitative difference between groups was accounted for by CSFC.

The individual tracts chosen represent the major temporal association pathways, which connect the temporal lobe to other cortical regions, most notably in frontal and parietal lobes. They have previously been shown to be compromised in MCI (Choo et al., 2010; Fujie et al., 2008; Metzler-Baddeley et al., 2012b; Mielke et al., 2009). Overall, the effect of FWE correction on disease-related changes across these pathways was to shift the apparent pattern of tract involvement as well as the profile of observed differences in terms of which DTI indices are most sensitive.

In the case of the fornix, the results suggest a true difference in FA that was attenuated by CSFC, while there was no difference between groups for MD after applying the FWE approach. This result fits with both the unique intraventricular location of the fornix and evidence that its intrinsic structure is altered as a result of Alzheimer pathology (Lee et al., 2012). Both analyses suggested altered microstructure of left and right PHC, but in this case CSFC seemed to exaggerate the effects of a true difference on FA measurements. The PHC was unusual in demonstrating *increased* FA in MCI compared to controls, and PVE tended to increase the difference between groups in this case.

The shifts in apparent spatial pattern of involvement are important as many studies continue to make interpretations about selective involvement of tracts based on diffusion MRI and tractography. It is important to appreciate that apparent selective involvement of some tracts might arise from their susceptibility to CSFC and other sources of measurement error and bias rather than any direct involvement in the disease process. Tracts differ in their vulnerability to CSFC – by virtue of their position relative to CSF spaces, size and shape – as well as in their vulnerability to pathology. For example, the uncinate is exposed to CSFC because of its close anatomical relationship to the Sylvian fissure. It is important that factors related to measurement and error, such as CSFC, are considered before assuming that a larger apparent effect in an individual tract denotes pathological vulnerability.

In some individual tract analyses, correcting for CSFC resulted in a larger change in the value of diffusion metric in the control than MCI group. This counterintuitive finding was not corroborated by the TBSS analysis, where comparison of difference images revealed no voxel where there was a significantly greater difference for controls. The reason for this observation at the individual tract level is not clear. One possibility is the influence of three-dimensional shape differences and possibly alignment with the underlying imaging matrix, though it seems unlikely that there was a systematic difference in the latter. Pinpointing an explanation is hampered by the fact that so many factors influence the appropriateness of the tensor model (bi-tensor model in this case) and the measurements that emerge. Diffusion approaches that better combine macrostructural and microstructural information (Assaf et al., 2013), and approaches to measure tissue water content independent of diffusion (Shah et al., 2011), could potentially aid with the interpretation of any idiosyncratic effects of FWE correction.

It has been argued that TBSS should be more robust to PVE than other voxel-based approaches as it assesses diffusion indices only in the estimated centres of white matter tracts (Smith et al., 2006). Smith et al. (2006) have argued that values of diffusion indices are unaffected by PVE in tracts that are wider than the relevant voxel dimension. This has been supported by *post mortem* high-resolution diffusion scanning, suggesting that, at typical resolutions obtained in vivo, tracts appear thinner due to PVE (Miller et al., 2011). Though feasible, this contention has not been tested systematically in real in vivo data. The current analysis suggests that this argument is largely true but with specific caveats. In particular, uncorrected TBSS analyses tended to exaggerate group differences in fornix microstructure (Figs. 1 and 2). In fact, this result is consistent with the criterion put forward by Smith et al., as in many cases the width of the fornix is not much greater than typical voxel dimensions of diffusion MRI acquisitions. However, the results do illustrate that some tracts that are typically included in the TBSS skeleton are not sufficiently large relative to voxel size to be immune from CSFC.

At a more subtle level, CSFC appeared to introduce a spatial bias to TBSS results. In particular, CSFC had a relatively stronger effect in the MCI group in the anterior corpus callosum and adjacent frontal white matter (Suppl. Fig. 2). This bias was reflected in maps of apparent effect size (Fig. 4). Effect size was represented by Cohen's *d* as this relates directly to the power to detect a significant difference in a group comparison based on *t*-tests. Apparent effect sizes varied spatially, between metrics, and between corrected and uncorrected data. These variations suggest that caution is needed in interpreting spatial patterns of results that emerge from applying fixed significance thresholds.

At a more subtle level, CSFC appeared to introduce a spatial bias to TBSS results. In particular, CSFC had a relatively stronger effect in the MCI group in the anterior corpus callosum and adjacent frontal white matter (Suppl. Fig. 2). This bias was reflected in maps of apparent effect size (Fig. 4). Effect size was represented by Cohen's *d* as this relates directly to the power to detect a significant difference in a group comparison based on *t*-tests. Apparent effect sizes varied spatially, between metrics, and between corrected and uncorrected data. These variations suggest that caution is needed in interpreting spatial patterns of results that emerge from applying fixed significance thresholds.

An analysis of whole white matter histograms was included because these measures have been advocated as useful summary statistics both for group studies and for longitudinal studies of disease progression (Chabriat, 2005). However, histograms are likely to be susceptible to CSFC because atrophy leads to an increase in surface area to volume ratio, increasing the relative contribution of voxels contaminated by CSF. Indeed, in previous studies that have used histograms a peripheral rim of voxels with higher diffusivity raises the possibility that CSF-contaminated voxels are being included in histograms (Jouvent et al., 2007). FWE had little effect on the presence of group differences but analysis of the relative group differences in summary statistics suggested that CSFC was a substantial contributor to many apparent differences. This contribution varied between measures: it was approximately 16% for group differences in mean FA and 33% for group differences in mean MD. This suggests different sensitivity of measures derived from histograms to volumetric effects. Strikingly, the ratio of percentage error for MD compared to FA matches the predictions based on simulations of CSFC on single voxels (Metzler-Baddeley et al., 2012a). Diffusivity histogram measures in the MCI group were affected disproportionately by CSFC, as indicated by significant interactions between histogram metrics and FWE correction. One explanation of the roughly equivalent group effect sizes for histogram mean and modal values is that histogram analysis of uncorrected data is partly driven by volumetric change, while histograms of corrected data have greater sensitivity to true intrinsic microstructural change. Regardless of the detailed explanation, measures derived from histograms should be interpreted with caution and considered a mixed measure of both intrinsic microstructural and volumetric factors.

The FWE approach employs a two-compartment approach to model the contributions of brain tissue and free water to the averaged intravoxel diffusion signal, assuming a fixed (high) diffusivity for the free water compartment. As such, it cannot correct for PVE due to signal from grey matter (where the mean diffusivity, at the b-values used in the current study, is comparable across white and grey matter), which is an additional potential source of bias in the studies of neurodegeneration. On a more technical note, the FWE approach involves a simple model of the signal attenuation: that of two non-interacting (non-exchanging) homogenous compartments, and adopts a simple bi-exponential model to characterize the system. It should be stressed that this is a simple model that is only valid when the diffusion weighting is sufficiently strong to suppress the acquired signal before the spins in each packet spread over distances that exceed their disorder correlation length (Novikov and Kiselev, 2010). The extent to which adopting a full effective medium theory approach (Novikov and Kiselev, 2010) actually impacts on the results obtained here is beyond the scope of the current work, and remains an open question for further consideration.

The results of this study demonstrate that CSF contamination influences the apparent tissue microstructural alterations in MCI observed in patient–control group studies. CSF contamination has quantitative effects at various spatial levels. In addition, it alters the apparent spatial pattern of involvement, particularly at the level of individual tracts which vary in their susceptibility. Tract-specific alterations in diffusion metrics are usually assumed to be due to alterations in intrinsic microstructure. These results illustrate that contamination through partial volume effects, influenced by macrostructural features such as tract size and shape should not be forgotten in the interpretation of diffusion MRI group studies.

The following are the supplementary data related to this article.

**Suppl. Fig. 1:**
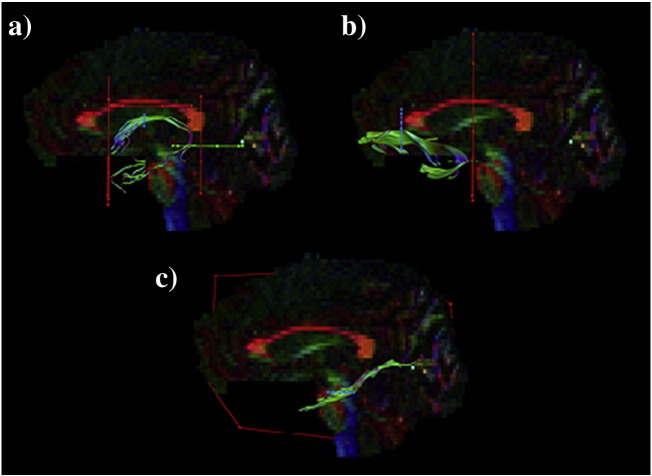
Reconstructions of temporal association tracts. Tractography using region of interest (ROI) waypoints (seedpoint ROIs depicted in blue, AND ROIs in green, NOT ROIs in red) in the native space of one participant for: a) fornix; b) uncinate fasciculus; c) parahippocampal cingulum.

**Suppl. Fig. 2:**
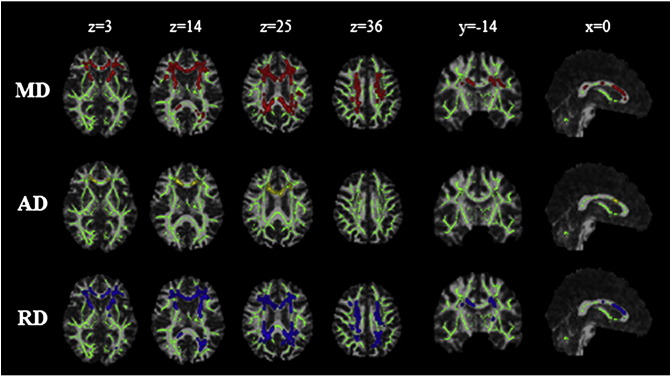
Group differences in the spatial pattern of CSF contamination. A group comparison of difference images was performed: areas showing significantly higher influence of CSF partial volume effect on diffusivity parameter values in MCI are highlighted in colour (corrected for family-wise error and thresholded for *p* < .05). Statistical maps are overlaid on the registration target brain transformed into MNI152 space.

Supplementary material.

Supplementary data to this article can be found online at http://dx.doi.org/10.1016/j.neuroimage.2014.01.031.

## Figures and Tables

**Table 1:** Comparison of individual tracts between MCI and controls, before and after correction with Free Water Elimination. Tract-specific average values (SD) of fractional anisotropy (FA, no unit) and mean diffusivity (MD, 10^− 3^ mm^2 ^s^− 1^) are provided. Examples where CSF contamination altered the qualitative interpretation of results are highlighted in bold. Abbreviations: UNC, uncinate fasciculus; PHC, parahippocampal cingulum; MCI, mild cognitive impairment; HC, healthy control; FWE, Free Water Elimination; *T* is the t-statistic and *p* the associated probability for a two-tailed *t*-test. *d* is Cohen's d.

		Group	Uncorrected				FWE-corrected			
			Mean (SD)	*T*^a^	*p*	*d*	Mean (SD)	*T*^a^	*p*	*d*
Fornix	FA	MCI	0.201 (0.027)	− 2.96	.005**	.89^+++^	0.237 (0.045)	− 2.82	.007**	
		HC	0.224 (0.023)				0.272 (0.037)			.85^+++^
	MD	MCI	**1.876 (0.238)**	**3.22**	.002**	**.97^+++^**	**1.020 (0.125)**	**− 1.54**	**.13**	
		HC	**1.660 (0.205)**				**1.065 (0.069)**			**.43^+^**
UNC left	FA	MCI	**0.401 (0.025)**	**2.85**	.007**		**0.429 (0.053)**	**0.79**	**.43**	
		HC	**0.377 (0.030)**			**.85^+++^**	**0.418 (0.029)**			**.24^+^**
	MD	MCI	**0.880 (0.048)**	**1.38**	**.18**		**0.802 (0.040)**	**2.11**	.041*	
		HC	**0.861 (0.043)**			**.41^+^**	**0.781 (0.027)**			**.61^++^**
UNC right	FA	MCI	0.395 (0.027)	1.91	.063		0.427 (0.056)	0.48	.63	
		HC	0.380 (0.023)			.57^++^	0.420 (0.025)			.15
	MD	MCI	**0.892 (0.036)**	**1.80**	**.078**		**0.816 (0.047)**	**2.33**	.025*	
		HC	**0.872 (0.035)**			**.54^++^**	**0.790 (0.023)**			**.70^++^**
PHC left	FA	MCI	0.342 (0.033)	3.44	.001**		0.386 (0.032)	3.43	.001**	
		HC	0.311 (0.023)			1.1^+^	0.356 (0.023)			1.0^+++^
	MD	MCI	0.845 (0.054)	− 0.042	.97		0.786 (0.040)	0.089	.93	
		HC	0.846 (0.042)			.01	0.785 (0.028)			.03
PHC right	FA	MCI	0.364 (0.037)	3.35	.002**		0.409 (0.033)	3.31	.002**	
		HC	0.331 (0.026)			1.0^+++^	0.379 (0.026)			1.0^+++^
	MD	MCI	0.827 (0.067)	− 0.12	.91		0.767 (0.042)	− 0.41	.69	
		HC	0.829 (0.048)			.04	0.772 (0.031)			.12

Significance: *p < .05, **p < .01. Effect sizes: + small (d > .2), ++ medium (d > .5), +++ large (d > .8).

a *df* = 43 (except for left and right PHC, where *df* = 41).

**Table 2:** Comparisons of summary statistics derived from histograms between MCI and control groups, before and after correction with Free Water Elimination. Mean and modal values of the histogram distribution for fractional anisotropy (FA, no unit), mean diffusivity (MD, 10^− 3^ mm^2 ^s^− 1^), axial diffusivity (AD, 10^− 3^ mm^2 ^s^− 1^) and radial diffusivity (RD, 10^− 3^ mm^2 ^s^− 1^) and corresponding histogram peak heights (proportion of voxels at modal value) for patients with mild cognitive impairment (MCI) and healthy elderly controls (HC) in the uncorrected dataset, and after correcting for partial volume effect using the free-water elimination (FWE) approach. Examples where CSF contamination altered the qualitative interpretation of results are highlighted in bold.

		Group	Uncorrected				FWE-corrected			
			Mean (SD)	*T*(*df* = 43)	*p*	*d*	Mean (SD)	*T*(*df* = 43)	*p*	*d*
FA	Mean	MCI	0.323 (0.029)	− 3.08	.004**		0.353 (0.028)	− 3.01	.005**	
		HC	0.345 (0.017)			.93^++^	0.373 (0.015)			.85^++^
	Mode	MCI	0.262 (0.039)	− 3.45	.001**		0.284 (0.062)	− 3.40	.002**	
		HC	0.300 (0.034)			1.0^++^	0.336 (0.038)			.97^++^
	Peak height	MCI	0.0145 (0.0015)	1.33	.19		0.0130 (0.0020)	0.95	.35	
		HC	0.0139 (0.0009)			.40^+^	0.0127 (0.0008)			.28
MD	Mean	MCI	0.871 (0.066)	2.35	.023*		0.780 (0.039)	2.31	.026*	
		HC	0.833 (0.033)			.70^+^	0.757 (0.021)			.69^+^
	Mode	MCI	**0.803 (0.040)**	**1.87**	**.069**		**0.753 (0.029)**	**2.04**	.048*	
		HC	**0.784 (0.030)**			**.56^+^**	**0.737 (0.020)**			**.61^+^**
	Peak height	MCI	0.0340 (0.0070)	− 3.76	.001**		0.0529 (0.0107)	− 3.66	.001**	
		HC	0.0411 (0.0053)			1.1^++^	0.0634 (0.0079)			1.1^++^
AD	Mean	MCI	1.166 (0.055)	1.64	.11		1.071 (0.029)	0.98	.33	
		HC	1.143 (0.031)			.49	1.064 (0.018)			.29
	Mode	MCI	1.068 (0.042)	0.84	.41		0.997 (0.031)	1.74	.088	
		HC	1.059 (0.029)			.25	0.983 (0.019)			.52^+^
	Peak height	MCI	0.0196 (0.0044)	− 2.58	.013*		0.0249 (0.0022)	− 2.35	.024*	
		HC	0.0223 (0.0014)			.77^+^	0.0262 (0.0013)			.70^+^
RD	Mean	MCI	0.723 (0.073)	2.77	.009**		0.634 (0.046)	2.70	.010*	
		HC	0.677 (0.035)			.78^+^	0.604 (0.024)			.81^++^
	Mode	MCI	**0.661 (0.045)**	**2.03**	.048*		**0.618 (0.033)**	**1.87**	**.069**	
		HC	**0.637 (0.033)**			**.61^+^**	**0.601 (0.025)**			**.60^+^**
	Peak height	MCI	0.0285 (0.0048)	− 3.45	.001**		0.0370 (0.0048)	− 3.32	.002**	
		HC	0.0326 (0.0027)			1.0^++^	0.0410 (0.0028)			1.0^++^

Significance: *p < .05, **p < .01. Effect sizes: + medium (*d* > .5), ++ large (*d* > .8).
