# Supplementary material for: CSF contamination contributes to apparent microstructural alterations in mild cognitive impairment
Source: Neuroimage. 2014 May 15;92(100):27–35. doi: 10.1016/j.neuroimage.2014.01.031 (PMC4010672; doi:10.1016/j.neuroimage.2014.01.031)
Supplement: Inline Supplementary Table S1 [file mmc1.docx]

**Suppl. Table 1.** **Comparison of individual tracts between MCI and controls, before and after correction with Free Water Elimination, with a two-way mixed-design ANOVA.** Tract-specific average values (SD) of fractional anisotropy (FA, no unit) and mean diffusivity (MD, 10^-3^ mm^2^s^-1^) are provided. Abbreviations: UNC, uncinate fasciculus; PHC, parahippocampal cingulum; MCI, mild cognitive impairment; HC, healthy control; FWE, Free Water Elimination; *F* is the F-test statistic and *p* the associated probability.

|  |  |  | **Uncorrected** | **FWE-corrected** | **Within-subjects contrast**  **(FWE)** | | **Within-subjects contrast**  **(FWExGroup)** | | **Between-subjects effect**  **(Group)** | |
| --- | --- | --- | --- | --- | --- | --- | --- | --- | --- | --- |
|  |  |  | **Mean (SD)** | **Mean (SD)** | ***F*** | ***p*** | ***F*** | ***p*** | ***F*** | ***p*** |
| **Fornix** |  |  |  |  |  |  |  |  |  |  |
|  | FA | MCI | 0.201 (0.027) | 0.237 (0.045) |  |  |  |  |  |  |
|  |  | HC | 0.224 (0.023) | 0.272 (0.037) | 183.788 | <.001*** | 4.243 | .046* | 8.546 | .006** |
|  | MD | MCI | 1.876 (0.238) | 1.020 (0.125) |  |  |  |  |  |  |
|  |  | HC | 1.660 (0.205) | 1.065 (0.069) | 315.976 | <.001*** | 10.249 | .003** | 6.816 | .012* |
| **UNC left** |  |  |  |  |  |  |  |  |  |  |
|  | FA | MCI | 0.401 (0.025) | 0.429 (0.053) |  |  |  |  |  |  |
|  |  | HC | 0.377 (0.030) | 0.418 (0.029) | 38.262 | <.001*** | 1.371 | .248 | 3.239 | .079 |
|  | MD | MCI | 0.880 (0.048) | 0.802 (0.040) |  |  |  |  |  |  |
|  |  | HC | 0.861 (0.043) | 0.781 (0.027) | 415.116 | <.001*** | .069 | .793 | 2.983 | .091 |
| **UNC right** |  |  |  |  |  |  |  |  |  |  |
|  | FA | MCI | 0.395 (0.027) | 0.427 (0.056) |  |  |  |  |  |  |
|  |  | HC | 0.380 (0.023) | 0.420 (0.025) | 35.287 | <.001*** | .441 | .510 | 1.319 | .257 |
|  | MD | MCI | 0.892 (0.036) | 0.816 (0.047) |  |  |  |  |  |  |
|  |  | HC | 0.872 (0.035) | 0.790 (0.023) | 220.592 | <.001*** | .450 | .506 | 5.580 | .023* |
| **PHC left** |  |  |  |  |  |  |  |  |  |  |
|  | FA | MCI | 0.342 (0.033) | 0.386 (0.032) |  |  |  |  |  |  |
|  |  | HC | 0.311 (0.023) | 0.356 (0.023) | 1331.118 | <.001*** | .029 | .865 | 12.050 | .001** |
|  | MD | MCI | 0.845 (0.054) | 0.786 (0.040) |  |  |  |  |  |  |
|  |  | HC | 0.846 (0.042) | 0.785 (0.028) | 453.435 | <.001*** | .029 | .866 | .001 | .970 |
| **PHC right** |  |  |  |  |  |  |  |  |  |  |
|  | FA | MCI | 0.364 (0.037) | 0.409 (0.033) |  |  |  |  |  |  |
|  |  | HC | 0.331 (0.026) | 0.379 (0.026) | 611.395 | <.001*** | .380 | .541 | 11.522 | .002** |
|  | MD | MCI | 0.827 (0.067) | 0.767 (0.042) |  |  |  |  |  |  |
|  |  | HC | 0.829 (0.048) | 0.772 (0.031) | 202.064 | <.001*** | .095 | .759 | .054 | .818 |

Significance: * *p* < .05, ** *p* <.01, *** *p* <.001
