# Supplementary material for: CSF contamination contributes to apparent microstructural alterations in mild cognitive impairment
Source: Neuroimage. 2014 May 15;92(100):27–35. doi: 10.1016/j.neuroimage.2014.01.031 (PMC4010672; doi:10.1016/j.neuroimage.2014.01.031)
Supplement: Inline Supplementary Table S2 [file mmc2.docx]

**Suppl. Table 2.** **Summary statistics derived from histograms of MCI and control groups and results of a two-way mixed-design ANOVA.** Mean and modal values of the histogram distribution for fractional anisotropy (FA, no unit), mean diffusivity (MD, 10^-3^ mm^2^s^-1^), axial diffusivity (AD, 10^-3^ mm^2^s^-1^) and radial diffusivity (RD, 10^-3^ mm^2^s^-1^) and corresponding histogram peak heights (proportion of voxels at modal value) for patients with mild cognitive impairment (MCI) and healthy elderly controls (HC) in the uncorrected dataset, and after correcting for partial volume effect using the free-water elimination (FWE) approach. *F* is the F-test statistic, *p* is the associated probability.

|  |  |  | **Uncorrected** | **FWE-corrected** | **Within-subjects contrast (FWE)** | | **Within-subjects contrast (FWExGroup)** | | **Between-subjects effect (Group)** | |
| --- | --- | --- | --- | --- | --- | --- | --- | --- | --- | --- |
|  |  | **Group** | **Mean (SD)** | **Mean (SD)** | ***F*** | ***p*** | ***F*** | ***p*** | ***F*** | ***p*** |
| **FA** | Mean | MCI | 0.323 (0.029) | 0.353 (0.028) | 1270.361 | <.001*** | 2.604 | .114 | 8.861 | .005** |
|  |  | HC | 0.345 (0.017) | 0.373 (0.015) |  |  |  |  |  |  |
|  | Mode | MCI | 0.262 (0.039) | 0.284 (0.062) | 38.348 | <.001*** | 1.948 | .170 | 12.089 | .001** |
|  |  | HC | 0.300 (0.034) | 0.336 (0.038) |  |  |  |  |  |  |
|  | Peak Height | MCI | 0.0145 (0.0015) | 0.0130 (0.0020) | 182.138 | <.001*** | 0.308 | .582 | 1.347 | .252 |
|  |  | HC | 0.0139 (0.0009) | 0.0127 (0.0008) |  |  |  |  |  |  |
| **MD** | Mean | MCI | 0.871 (0.066) | 0.780 (0.039) | 620.202 | <.001*** | 5.536 | .023* | 5.471 | .024* |
|  |  | HC | 0.833 (0.033) | 0.757 (0.021) |  |  |  |  |  |  |
|  | Mode | MCI | 0.803 (0.040) | 0.753 (0.029) | 624.846 | <.001*** | 1.220 | .275 | 3.823 | .057 |
|  |  | HC | 0.784 (0.030) | 0.737 (0.020) |  |  |  |  |  |  |
|  | Peak Height | MCI | 0.0340 (0.0070) | 0.0529 (0.0107) | 1662.902 | <.001*** | 10.848 | .002** | 13.767 | .001** |
|  |  | HC | 0.0411 (0.0053) | 0.0634 (0.0079) |  |  |  |  |  |  |
| **AD** | Mean | MCI | 1.166 (0.055) | 1.071 (0.029) | 638.238 | <.001*** | 4.909 | .032* | 2.016 | .163 |
|  |  | HC | 1.143 (0.031) | 1.064 (0.018) |  |  |  |  |  |  |
|  | Mode | MCI | 1.068 (0.042) | 0.997 (0.031) | 712.148 | <.001*** | .633 | .431 | 1.569 | .217 |
|  |  | HC | 1.059 (0.029) | 0.983 (0.019) |  |  |  |  |  |  |
|  | Peak Height | MCI | 0.0196 (0.0044) | 0.0249 (0.0022) | 125.521 | <.001*** | 2.769 | .103 | 7.596 | .009** |
|  |  | HC | 0.0223 (0.0014) | 0.0262 (0.0013) |  |  |  |  |  |  |
| **RD** | Mean | MCI | 0.723 (0.073) | 0.634 (0.046) | 607.273 | <.001*** | 5.377 | .025* | 6.927 | .012** |
|  |  | HC | 0.677 (0.035) | 0.604 (0.024) |  |  |  |  |  |  |
|  | Mode | MCI | 0.661 (0.045) | 0.618 (0.033) | 397.796 | <.001*** | 3.808 | .058 | 3.899 | .055 |
|  |  | HC | 0.637 (0.033) | 0.601 (0.025) |  |  |  |  |  |  |
|  | Peak Height | MCI | 0.0285 (0.0048) | 0.0370 (0.0048) | 3523.479 | <.001*** | .149 | .702 | 11.606 | .001** |
|  |  | HC | 0.0326 (0.0027) | 0.0410 (0.0028) |  |  |  |  |  |  |

Significance: * *p* < .05, ** *p* <.01, *** *p* <.001
