# Supplementary material for: CSF contamination contributes to apparent microstructural alterations in mild cognitive impairment
Source: Neuroimage. 2014 May 15;92(100):27–35. doi: 10.1016/j.neuroimage.2014.01.031 (PMC4010672; doi:10.1016/j.neuroimage.2014.01.031)
Supplement: Supplementary file 3 — Supplementary material. [file mmc3.docx]

*Supplementary methods*

Tractography reconstructions were performed using multiple waypoint regions of interest (ROIs) based on anatomical landmarks. SEED regions defined the starting points of the tractography algorithm. Of streamlines emanating from the SEED regions only those passing through AND ROIs but not traversing NOT ROIs were retained (Suppl. Fig. 1).

*Fornix*

A seed point ROI was placed medially on a coronal slice around the fornix bundle at the level of the entry point of the anterior pillars into the body of the fornix, approximately below the sagittal midline of the corpus callosum. An AND ROI was defined on an axial slice capturing the crus fornici in both hemispheres at the level of the inferior border of the splenium of the corpus

callosum. NOT ROIs were drawn on coronal slices rostral to the anterior fornix pillars and caudal to the crus fornici as well as on axial slices through the corpus callosum and the upper pons to exclude streamlines from the corpus callosum and the corticospinal tract. Anatomically implausible outlier streams, if present, were excluded using additional NOT ROIs.

*Uncinate fasciculus (UNC)*

A seed point ROI was drawn on a coronal slice around the region where the UNC enters the frontal lobe immediately rostral to the genu of the corpus callosum. An AND ROI was placed on an axial slice capturing the UNC bundle at the point where the bundle bends into the inferior temporal lobe region. This bend was visually identified on the midline sagittal plane with the axial slice being placed at a level immediately dorsal to the upper pons. A NOT ROI was drawn across the coronal slice level with the front of the pons to exclude tracts of the inferior fronto-occipital fasciculus. Any obvious outlier streamlines that were inconsistent with the known UNC anatomy were excluded using additional NOT ROIs. This procedure was repeated for the left and right UNC.

*Parahippocampal cingulum (PHC)*

A seed point ROI was placed on an axial slice level with the pons–midbrain junction around the cingulum fiber bundle. A NOT ROI was drawn across the midline sagittal plane to remove interhemispheric projections. After visual inspection, further NOT ROIs were placed, when necessary, to exclude projections to the occipital lobe and any outlier tracts that were not consistent with the known anatomy of the PHC. This procedure was performed separately for the left and the right PHC.
